# Epithelioid angiosarcoma of the kidney: A case report and literature review

**DOI:** 10.3892/ol.2014.2292

**Published:** 2014-06-26

**Authors:** HONGYUN LIU, XINGANG HUANG, HUA CHEN, XUECHUN WANG, LEI CHEN

**Affiliations:** 1 Department of Pathology, Municipal Hospital, Qingdao University Medical College, Qingdao, Shandong 266071, P.R. China; 2 Department of Radiology, Municipal Hospital, Qingdao University Medical College, Qingdao, Shandong 266071, P.R. China; 3 Department of Physiology, Qingdao University Medical College, Qingdao, Shandong 266071, P.R. China

**Keywords:** angiosarcoma, kidney, epithelioid, sarcoma

## Abstract

Epithelioid angiosarcoma (EAS) is a rare disease which presents a great diagnostic challenge. The present study reports a case of EAS in the kidney in a 75-year-old male who presented with gross hematuria. An abdominal computed tomography scan revealed space-occupying lesions of the right kidney and renal cell carcinoma was suspected. Histological examination of the resected specimens showed pleomorphic epithelioid cells with vesicular nuclei, prominent nucleoli and eosinophilic cytoplasm that lined irregular vascular spaces. Immunohistochemical staining revealed that the tumor cells were positive for AE1/AE3, cytokeratin (CK) 7, vimentin, cluster of differentiation (CD) 31 and E-cadherin, but showed no staining for CD10, CD34, factor VIII, CK20, carcinoembryonic antigen or desmin. Based on the histopathological and immunohistochemical findings, the patient was diagnosed with epithelioid angiosarcoma. Postoperative radiation therapy was administered and no recurrence was observed six months after surgery.

## Introduction

Angiosarcomas are rare, high-grade malignancies of endothelial origin. The rarity of angiosarcomas makes clinical diagnosis difficult. Histologically, angiosarcomas range from well-differentiated tumors with variable endothelial atypia to high-grade spindle cell neoplasms. Unlike the conventional appearance, a particular morphological subtype of angiosarcoma, in which the neoplastic endothelial cells have a predominantly epithelioid character, has been termed epithelioid angiosarcoma (EAS) (1). EAS most often arises in the deep soft tissues of the extremities (2), but a variety of primary sites, including the thyroid gland, skin, adrenal glands, gallbladder (3), uterus (4), tonsil (5) and bone, have been reported (6). The pathological features of primary renal EAS have been previously described in a study which used fine-needle aspiration cytology (7). The present study reports a case of EAS of the kidney that was diagnosed using resected tissue sections. Based on its site of origin and the histological images, it is necessary to distinguish EAS from other tumors of the kidney, including metastatic carcinoma, melanoma and other epithelioid and rhabdoid neoplasms. Immunohistochemical findings are useful for diagnosing EAS. The patient provided written informed consent.

## Case report

### Patient presentation

**Figure 1:**
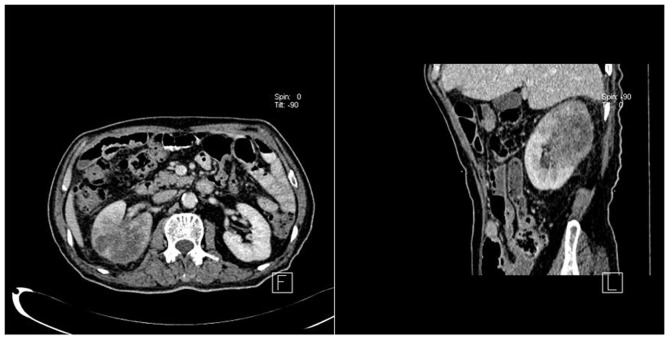
Abdominal computed tomography scan showing a large mass in the upper-mid portion of the right kidney with mixed density.

A 75-year-old male presented at the Municipal Hospital (Qingdao, China) with the chief complaint of recurrent gross hematuria for approximately one month. An abdominal computed tomography (CT) scan revealed space-occupying lesions in the upper-mid section of the right kidney with mixed density (Fig. 1). No lesions were identified in the other abdominal viscera or soft tissues. Renal cell carcinoma (RCC) was suspected and the right kidney was resected.

### Pathological findings

**Figure 2:**
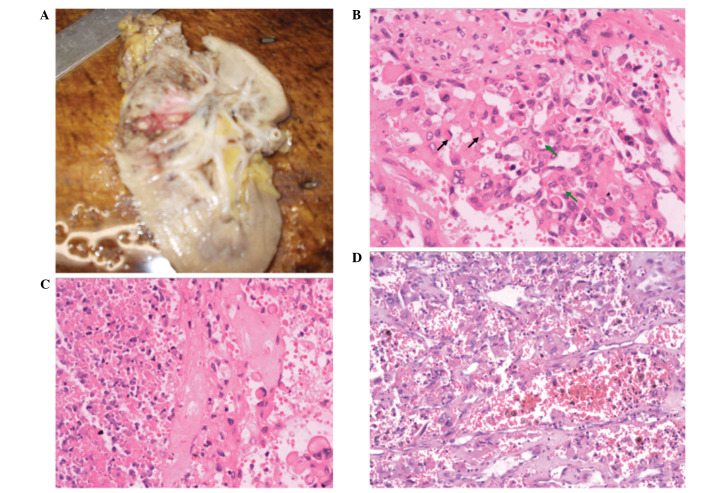
(A) The kidney tumor appears as a white and light-brown neoplasm with poorly defined margins and partial necrosis. (B) H&E-stained section demonstrating the proliferation of large, pleomorphic cells with abundant eosinophilic cytoplasm and pleomorphic nuclei. Certain cells mimicked signet ring cells (black arrows) and others had an intracytoplasmic lumina containing erythrocytes (green arrows). Magnification, ×400. (C) Atypical cells are arranged in sheets with extensive necrosis (left part). H&E staining; magnification, ×400. (D) A complex network of vascular-like spaces within the tumor. H&E staining; magnification, ×100. H&E, hematoxylin & eosin.

**Figure 3:**
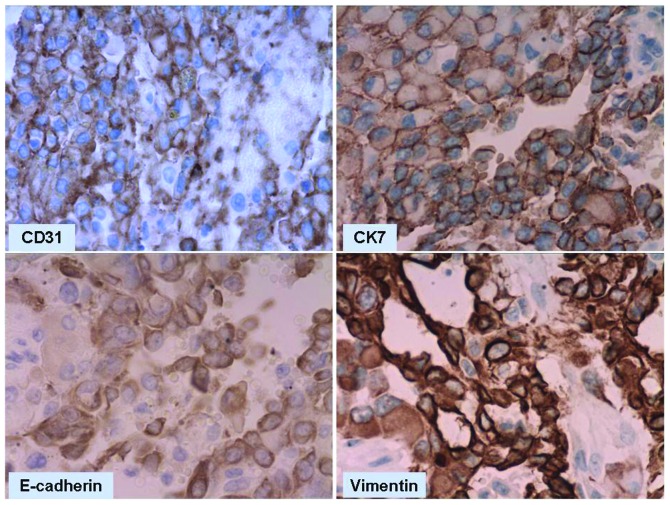
Immunohistochemical staining showing that the tumor cells were positive for CD31, CK7, E-cadherin and vimentin. Magnification, ×400. CD, cluster of differentiation; CK, cytokeratin.

Grossly, the tumors presented as a large, necrotic, hemorrhagic mass in the upper-mid section of the right kidney and measured 4×4 cm in diameter (Fig. 2A). The poorly defined tumor margins extended close to the renal capsule. Histopathology revealed large, mild-moderate pleomorphic, round-polygonal epithelioid cells or spindle cells, with vesicular nuclei containing prominent nucleoli. The malignant endothelial cells were found to be filled with abundant eosinophilic cytoplasm and certain cells appeared to mimic signet ring cells. A few cells were observed to have intracytoplasmic lumina containing erythrocytes, aiding the diagnosis (Fig. 2B). The cells were arranged in sheets with extensive necrosis (Fig. 2C). Focal areas of irregularly anastomosing vessel formation were also present (Fig. 2D). Immunohistochemistry was positive for AE1/AE3, cytokeratin (CK) 7, vimentin, cluster of differentiation (CD) 31 and E-cadherin (Fig. 3); however, no expression of CD10, CD34, factor VIII, CK20, carcinoembryonic antigen (CEA) or desmin was found. Staining for Ki-67 with MIB-1 was ~30%, confirming the highly proliferative nature of these neoplasms.

## Discussion

EAS has a male predilection and generally occurs in adults, with the highest incidence in individuals in their seventh decade (2). A number of clinical presentations may be encountered due to diversity in the primary sites of EAS. However, EAS of the kidney is particularly rare and few clinical features have been described. In the present case, the disease had developed insidiously and gross hematuria was the only clinical manifestation. A CT scan revealed a space-occupying lesion in the right kidney; however, the radiographic distinction between RCC, AS and EAS is complex, as all of these neoplasms are highly vascular and have large areas of necrosis (2).

The diagnosis of EAS primarily relies on pathological examination. Brown *et al* (8) reported and reviewed 25 cases of renal AS. Histologically, renal AS shows features similar to AS at other sites. Renal AS is often poorly differentiated, thus the diagnosis of renal AS requires the use of immunohistochemical methods to distinguish it from other renal tumors that also have prominent vascularity. Renal AS is positive for endothelial cell markers, including CD31, CD34 and factor VIII, but negative for the epithelial markers Cam 5.2, AE1/AE3 and EMA. A unique type of AS, termed EAS has not been described in previous studies, with the exception of a study which reported fine-needle aspiration cytology of primary renal EAS (2). The present study reported a case of EAS in the kidney which was diagnosed using resected tissue sections. In previous cases, immunostaining for factor VIII has been consistently positive among cases of EAS, with stronger staining observed in malignant cells compared with non-epithelioid vascular sarcomas (9). CD34 positivity has been reported to range between 40 and 100%, and it typically stains in areas with high vessel formation (9). In the present case, the epithelioid tumor cells were found to be positive for CD31, but negative for CD34 and factor VIII. Consequently, CD31 may be the most sensitive marker for identifying the poorly differentiated endothelial cells. EAS is difficult to diagnose using hematoxylin and eosin (H&E)-stained sections. In certain cases, a sheeted epithelioid appearance and positive CK staining make metastatic or primary carcinoma a strong diagnostic consideration. Distinguishing features that are observed using H&E analysis include areas of intracellular lumina, which may or may not contain erythrocytes, revealing the endothelial nature of EAS. The present case showed positive staining for AE1/AE3 and CK7, but no staining for CK20 or CEA. Of note, the tumor cells were found to be positive for E-cadherin which, to the best of our knowledge, has not been previously reported in AS. With the exception of primary and metastatic carcinoma, melanoma, malignant mesothelioma, anaplastic large cell lymphoma, epithelioid sarcoma, epithelioid hemangioendothelioma and malignant peripheral nerve sheath tumor may exhibit histologic characteristics similar to EAS. These tumors may be differentiated using a panel of immunohistochemical stains (10–12).

In conclusion, the present study has reported a case of EAS in the kidney and has reviewed findings from previous studies. Although these soft tissue lesions most commonly occur at other sites within the body, it is important that pathologists are aware that they may also occur in the kidney. In the present case, follow-up data suggested that early diagnosis may enhance survival.
